# Frailty phenotypes and their association with health consequences: a comparison of different measures

**DOI:** 10.1007/s40520-024-02887-4

**Published:** 2024-12-03

**Authors:** Yu-Chun Lin, Huang-Ting Yan

**Affiliations:** 1 Department of Chinese Medicine, China Medical University Hospital, Taichung, Taiwan; 2 Graduate Institute of Integrated Medicine, College of Chinese Medicine, China Medical University, Taichung, Taiwan; 3 Institute of Political Science, Academia Sinica, 128 Academia Rd., Sec.2, Nankang, 115, Taipei, Taiwan, ROC

**Keywords:** Comorbidity, Depression, Fractures, Frailty, Hospitalization

## Abstract

**Objectives:**

The frailty index is widely used in clinical and community settings to assess health status. This study aimed to identify the potential phenotypes of frail older adults and examine their relationship with health consequences compared with existing frailty measures.

**Methods:**

The 11-year follow-up data from the Taiwan Longitudinal Study on Aging, covering 5,334 individuals aged ≥ 50 years, were analyzed using random-effects panel logit models. We identified three frailty phenotypes: energy-based frailty (EBF), sarcopenia-based frailty (SBF), and hybrid-based frailty (HBF). Existing frailty measures such as the Study of Osteoporotic Fractures (SOF), Fatigue, Resistance, Ambulation, Illness, and Loss of weight (FRAIL), and Fried scales were applied. We examined their correlation with health outcomes, such as falls and fractures, depression, comorbidities, hospitalization, emergency department visits, and mortality, adjusting for individual-level characteristics.

**Results:**

Individuals with only EBF were found to be at a lower risk of falls and fractures than their counterparts with only SBF (adjusted odds ratio [AOR] = 0.13, 95% confidence interval [CI] = 0.03–0.46). Depression was less likely in the SBF group than in the EBF group (AOR = 0.02, 95% CI = 0.01–0.05). Hybrid-based frail older adults were more likely to be hospitalized (AOR = 1.84, 95% CI = 1.08–3.14) and have emergency department visits (AOR = 2.03, 95% CI = 1.15–3.58). Frailty assessed using existing measures was associated with adverse health outcomes.

**Conclusion:**

The proposed frailty phenotype classification differs from the existing frailty measures in its ability to distinguish the corresponding phenotypes underlying various health consequences. Governments may develop strategies based on frailty phenotypes to mitigate adverse health consequences.

**Supplementary Information:**

The online version contains supplementary material available at 10.1007/s40520-024-02887-4.

## Introduction

Frailty is a common geriatric syndrome characterized by an age-dependent decrease in functional reserves involving multiple organs and systems, resulting in the loss of biological reserves, increased vulnerability to stressors [1], and a higher risk of adverse health outcomes [2]. The estimated global prevalence of frailty among individuals aged ≥ 50 years is 12–24%, depending on the frailty scale used [3].

Frailty can be roughly divided into physical [4–8], cognitive, and social [9–11]. Researchers have developed a variety of indices to assess physical frailty, including the Frailty Index [FI] scale [4], the Clinical Frailty Scale [CFS] [5], the Fried index [6], the Study of Osteoporotic Fractures [SOF] index [7], and the Fatigue, Resistance, Ambulation, Illness and Loss of weight [FRAIL] index [8], among others. In general, frailty, defined by FI, FRAIL, or Fried, is associated with adverse health outcomes such as loss of activities of daily living, falls and fractures, depression, hospitalization, and mortality [12–19].

Components of the physical frailty scale can be grouped, and three areas of investigation are of particular significance. One study employed the FI scale to identify individuals who were multi-frail, cognitively and functionally frail, psychologically frail, and physiologically frail. This was followed by examining socioeconomic factors, immunosenescence markers, and inflammatory biomarkers associated with distinct frailty subtypes [20, 21]. Another study utilized the concept of health deficit reference to the FI to develop a continuous frailty scale, which was further divided into several groups ranging from robust to severe frailty. Those who are severely frail are most likely to have a high risk of poor health performance [22–24].

The other avenue of inquiry centers on the five phenotypic criteria proposed by Fried, which are intended to distinguish two [25] or three distinct subgroups [26, 27]. The MacArthur Study of Successful Aging captured two subdimensions of the Fried phenotype, in which slower gait, weaker grip strength, and lower physical activity define the first component that can better predict cognitive impairment, disability, and mortality, and exhaustion and weight loss define the second component [25]. The I-Lan Longitudinal Aging Study and the National Institute for Longevity Sciences-Longitudinal Study of Aging distinguished between three subgroups: non-mobility-type, mobility-type frailty, and low physical activity. They confirmed that the mobility subtype was associated with significantly adverse outcomes [26, 27]. Recent research compared the longitudinal trajectories of distinct pre-frailty (PF) groups based on the Fried frailty components, finding that the PF2, defined by one or two amongst weakness, slowness, and low physical activity, in the absence of exhaustion and unexplained weight loss, had a higher risk of having difficulties with instrumental activities of daily living and mortality than PF1, defined by exhaustion and/or unexplained weight loss, in the absence of weakness, slowness and low physical activity [28, 29].

These two points merit further investigation. First, the current studies solely classified frailty subtypes based on the components of a single frailty scale. However, there was some overlap in the components of some frailty scales. The SOF, Fried, and FRAIL scales assess involuntary body weight loss and fatigue. Among the remaining components, in addition to the number of ailments, most pertained to the indicators of muscle function and physical activity. Evidence suggests that decreased energy intake is linked to the loss of body fat, lean muscle tissue, and exhaustion [30]. Moreover, measures such as resistance (SOF and FRAIL), handgrip strength (Fried), walking ability (Fried and FRAIL), and physical activity (Fried) were used to evaluate muscle strength and function, as well as physical performance, all of which are closely associated with indicators of sarcopenia [31]. These results indicate that at least two types of frailty can be distinguished: energy- and sarcopenia-based. Second, most existing studies focusing on frailty subtypes have confirmed that one type performs worse than another in terms of health performance. The diverse aspects of health outcomes have not been discussed. We aimed to determine whether it was possible to create a classification for frailty that could explain the differences in various aspects of health consequences.

The present study employed panel data covering 5,334 individuals aged ≥ 50 in four waves from 1996 to 2007 to identify potential phenotypes of frail older adults and examine their relationship with health consequences compared to existing frailty measures.

## Methods

### Study Population

The unit of analysis was ‘individuals-year.’ This study used data collected from the Taiwan Longitudinal Study on Aging (TLSA), conducted by the Health Promotion Administration of Taiwan, thus offering a nationwide representative sample of the Taiwanese population. This longitudinal study was based on a three-stage equal-probability sampling design, using household registration data and information collected during face-to-face interviews. Data were collected across six waves from 1989 to 2007. We collected a sample of 12,626 pooled time-series and cross-sectional observations from four of the six survey rounds (1996, 1999, 2003, and 2007).

### Dependent variables

#### Fractures

The fractures were assessed using self-reported data. These data were based on two questions: ‘Has a doctor diagnosed you with a hipbone fracture?’ and ‘Are you taking medication or receiving treatment for a hipbone fracture?’ Hipbone fractures were identified only if patients were informed and treated (or medicated) by a healthcare professional. A dummy variable was created such that 1 = *respondents with hipbone fracture* and 0 = *otherwise*.

#### Depression

Depression was assessed utilizing a total of 10 items from the Center for Epidemiologic Studies Depression Scale (CES-D). Respondents were asked how frequently each item had been applied to during the preceding week. The ratings were based on a 4-point scale ranging from 0 (rarely or none of the time) to 3 (most or all of the time). Thus, this condensed version of the CES-D yields total individual scores ranging from 0 to 30, with higher scores indicating more depressive symptoms. A dummy variable was created such that 1 = *respondents with depression* (≥ 10 points) and 0 = *otherwise* (0–9 points) [32].

#### Comorbidity

Chronic diseases (e.g., hypertension, diabetes, heart disease, stroke, cancer, lung disease, arthritis, gastrointestinal disease, hepatobiliary disease, renal disease, and gout) were recorded using self-reported data. Several conditions were created based on the total number of chronic conditions for each participant (ranging from 0 to 11), and the number of morbidities was treated as a categorical variable (0, *absence of disease*; 1, *mild*; 2, *moderate*; ≥3, *severe*).

#### Hospitalization and emergency department (ED) visits

There were two self-reported questions. Hospitalization was measured by asking, ‘Were you admitted to a hospital in the past year?’ If the participants answered ‘yes,’ they were considered part of the group that had been hospitalized at least once. Emergency department (ED) visits were assessed by asking, ‘Did you visit the emergency department in the past year?’ If the participants answered ‘yes,’ they were considered part of the group with at least one ED visit.

#### Mortality

This study tracked whether participants survived or died during each subsequent wave of follow-up data and, if so, the year in which they passed away. Thus, the dependent variable was the failure or survival time of the individual (years).

### Independent variables

Three frailty phenotypes have been identified: energy-based frailty (EBF), sarcopenia-based frailty (SBF), and hybrid-based frailty (HBF). The measurements used to characterize the frailty phenotype were operationalized as follows:

#### Energy-based frailty: the EBF index

- Weight loss was defined as unintentional weight loss of at least 3 kg of body weight in the previous year.
- Fatigue/exhaustion was measured using the self-reported health question (‘In the past week, do you feel that you are unable to gather your energy to do things?’) and was categorized as 0 (‘no’ answer) or 1 (‘yes’ answer).

Participants were considered ‘energy-based frail’ if they fulfilled two criteria, ‘energy-based pre-frail’ if they fulfilled one criterion, and ‘robust’ if no criterion was fulfilled.

#### Sarcopenia-based frailty: the SBF index

- Low resistance was assessed by asking whether the participants encountered any difficulties in squatting. It was categorized as 0 (‘no difficulty’) or 1 (‘some difficulty, great difficulty, or cannot do it at all’).
- Low handgrip strength was evaluated by asking whether the participants had difficulty using their fingers to grasp or turn objects. Participants who had difficulty performing the test were considered to have low handgrip strength.
- Low walking ability was determined by asking whether the participants had difficulty walking 200–300 m. Participants who had difficulty performing the walking test or reported using a wheelchair were classified as having low mobility.
- Low physical activity was assessed by the incidence and progression of basic activities of daily living disability using the following question: ‘In the last year, did you have any deterioration in activities of daily living (bathing, dressing and undressing, feeding, getting out of bed, standing up, sitting on a chair, walking, and toileting)?’. Participants who had difficulty performing at least one of these activities were considered physically inactive.

Participants were considered ‘sarcopenia-based frail’ if at least three of the four criteria were fulfilled, ‘sarcopenia-based pre-frail’ if one or two criteria were present, and ‘robust’ if none were met. The four components of the SBF index assess upper limb muscle strength (*low handgrip strength*), lower limb muscle strength (*low resistance*), physical function (*low walking ability*), and physical activity (*low physical activity*).

#### Hybrid-based frailty

Hybrid-based frail older adults were defined as those who exhibited energy- and sarcopenia-based frailties. Thus, participants were considered ‘hybrid-based frail’ if they met two criteria of the EBF index and at least three of the four criteria for the SBF index. For comparison, we created a categorical variable for specific types of frailty: 0 = *robust and per-frailty*, 1 = *those who were exclusively energy-based frail*, 2 = *those who were exclusively sarcopenia-based frail*, and 3 = *those who were hybrid-based fail*.

#### Existing measures of frailty

This study employs the SOF (7), Fried (6), and FRAIL indices (8). Certain assessment questions were modified. The SOF index consists of three components: weight loss (the involuntary loss of 3 kg of body weight in the past year), chair stands (difficulty squatting), and reduced energy level (self-perceived reduced energy level as described by an answer of ‘yes, often or chronically’ to the question ‘In the past week, do you feel that you are unable to gather your energy to do things?’). Participants were considered ‘frail’ if at least two criteria were met.

The Fried index comprises five components: weight loss (the involuntary loss of 3 kg of body weight in the past year), fatigue/exhaustion (an answer of ‘yes, often or chronically’ to the question ‘In the past week, do you feel that you are unable to gather your energy to do things?’), weakness (difficulty in grasping or turning objects), slowness (difficulty walking 200–300 m), and low physical activity (difficulty performing at least one of the following activities: bathing, dressing and undressing, feeding, getting out of bed, standing up, sitting on a chair, walking, and toileting). Participants were considered ‘frail’ if three or more of these criteria were met.

The FRAIL index comprises five components: fatigue (an answer of ‘yes, often or chronically’ to the question ‘In the past week, do you feel that you are unable to gather your energy to do things?’), resistance (an answer of ‘yes’ to the question ‘do you have any difficulty doing the activities listed below by yourself? Walk up two or three flights of stairs’), ambulation (an answer of ‘yes’ to the question ‘do you have any difficulty doing the activities listed below by yourself? Walk for 200 to 300 meters’), illness (participants reported taking medication or receiving treatment for four or more of the following chronic diseases: hypertension, diabetes, heart disease, stroke, cancer, lung disease, arthritis, and renal disease), and weight loss (participants reported losing more than 3 kilograms of body weight during the last year). Participants were considered ‘frail’ if three or more of these criteria were met.

### Control variables

Individual-level characteristics were set as control variables, including sex (binary, 1 = *male*), age, education level (*no formal education: 0*,* elementary school: 1–6*,* junior to senior high school: 7–12*, and *college or above: ≥ 13 years*), area of residence (large city = 1, big city = 2, small city = 3, township = 4, and rural area = 5, with options 1, 2, and 3 were combined into ‘*urban*,’ options 4 were ‘*suburban*,’ and options 5 were ‘*rural*’*)*, marital status (binary, 1 = *married or living with a partner*), current living status (binary, 1 = *living alone*), smoking status (binary, 1 = *smoker*), alcohol intake (binary, 1 = *alcohol drinker*), and frequency of exercise (*0*,* 1*,* 2*, and *≥ 3 times per week*). Sociodemographic characteristics [12–19, 33, 34] and lifestyle factors [35–37] have been associated with health outcomes. Table S1 lists the characteristics of the sample.

### Statistical analysis

Statistical analyses were performed using Stata software. Descriptive statistics were used to describe the data, including absolute and percentage frequency distributions, means, and standard deviations. The Chi-square test and one-way analysis of variance (ANOVA) were used to compare categorical and continuous variables, with *p* < 0.05 indicating statistical significance. Each variable was evaluated separately in relation to the frailty phenotype.

The study used panel data from 5,334 people aged ≥ 50 across three waves from 1996 to 2007, yielding a sample of pooled time series and cross-sectional observations. Several fixed-effects and random-effects models can be employed with panel data depending on various factors. The standard errors from the fixed-effects models may be too large to tolerate if there is limited variability within subjects, as they require within-subject variations in the variables. Random-effects models are better suited for this scenario. Most older adults changed little over time; therefore, this study used a random-effects panel logit model.

The Kaplan-Meier (KM) method was used to graph survival curves, and the log–rank test was used to evaluate whether the KM curves for two or more groups were statistically equivalent. This study employed the Cox proportional hazards (PH) model to explain disparities in survival rates among older adults with distinct frailty phenotypes. The Cox PH model is robust and ensures that the results closely resemble those obtained from the correct parametric model. This is a safe choice because users must not worry about selecting the incorrect parametric model. When the PH assumptions are not met, this analysis introduces time-dependent covariates. This study considered this effect by multiplying it by time or some function of time, the choice of which was judged by graphical approaches or using the stratified Cox procedure for a single predictor that did not satisfy the PH assumption.

### Robustness tests

First, we employed an alternative measure of lower limb muscle strength (low resistance) to determine the SBF index. This was accomplished by asking participants if they had difficulty walking up two or three flights of stairs without using aids. Second, this study replaced one component of the EBF index with a component of the SBF index and vice versa. This study aimed to evaluate whether modified versions of the EBF and SBF scales could predict disparities in hipbone fractures and depression among older adults. If the disparate scales that we initially defined were capable of capturing these differences, their modified versions should not exhibit the same patterns in the prediction of hipbone fractures and depression. Otherwise, revising the components of the proposed index is imperative.

## Results

**Fig. 1:**
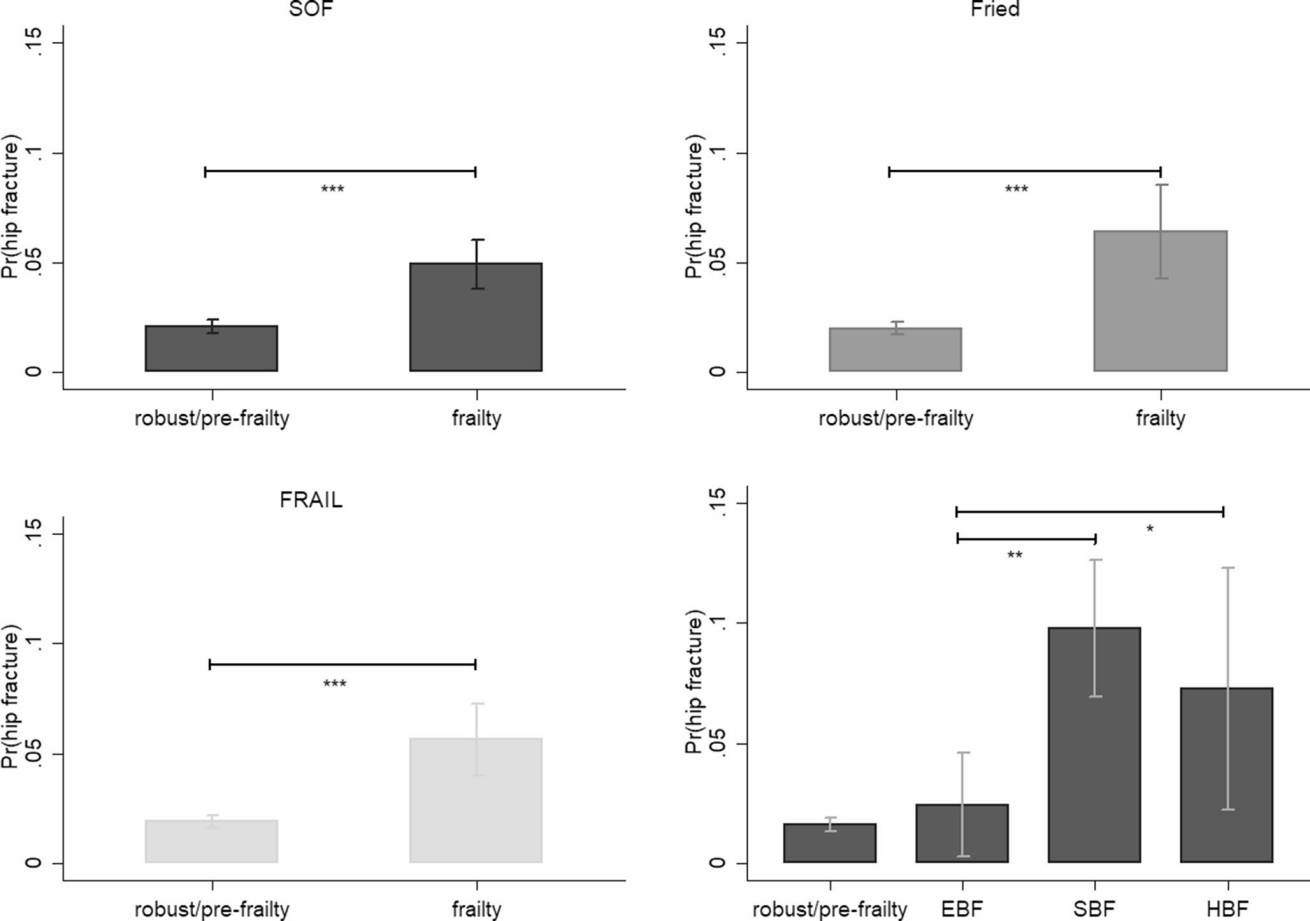
The effects of frailty and frailty phenotypes on the probability of fractures among middle-aged and older adults, Taiwan, 1996–2007. *Note* All results were based on random-effects panel logit model. The data points represent the mean ± standard error. Pr(hip fracture): the predicted probability of hip fracture; SOF: the Study of Osteoporotic Fractures index; Fried: the Fried’s frailty index; FRAIL: the Fatigue, Resistance, Ambulation, Illness and Loss of weight index; EBF: energy-based frailty; SBF: sarcopenia-based frailty; HBF: hybrid-based frailty; ^*^p < 0.05, ^**^p < 0.01, and ^***^p < 0.001. *Source*: the author

**Fig. 2:**
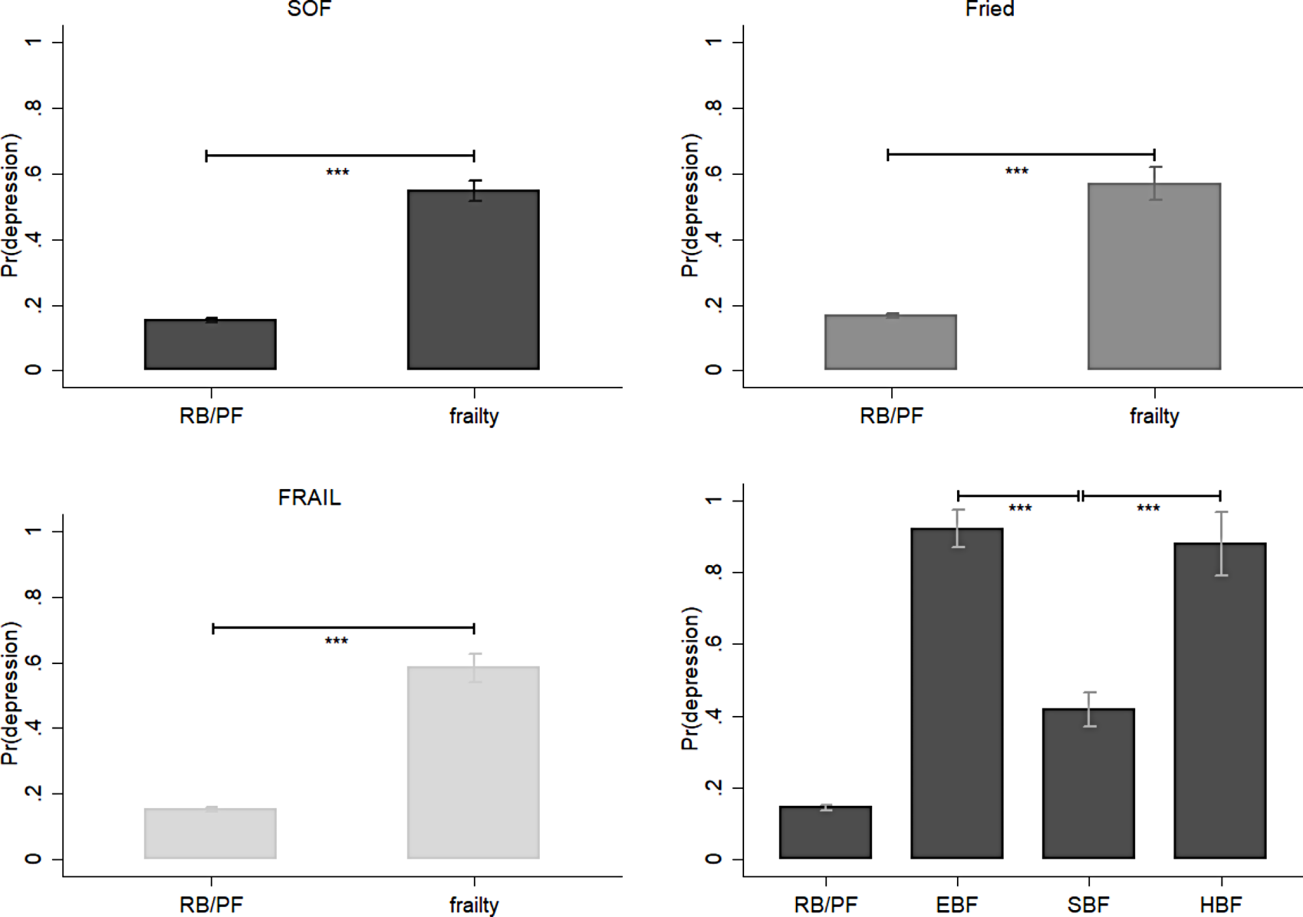
The effects of frailty and frailty phenotypes on the probability of depression among middle-aged and older adults, Taiwan, 1996–2007. *Note* All results were based on random-effects panel logit model. The data points represent the mean ± standard error. Pr(depression): the predicted probability of depression; SOF: the Study of Osteoporotic Fractures index; Fried: the Fried’s frailty index; FRAIL: the Fatigue, Resistance, Ambulation, Illness and Loss of weight index; EBF: energy-based frailty; SBF: sarcopenia-based frailty; HBF: hybrid-based frailty; ^*^p < 0.05, ^**^p < 0.01, and ^***^p < 0.001. *Source*: the author

**Fig. 3:**
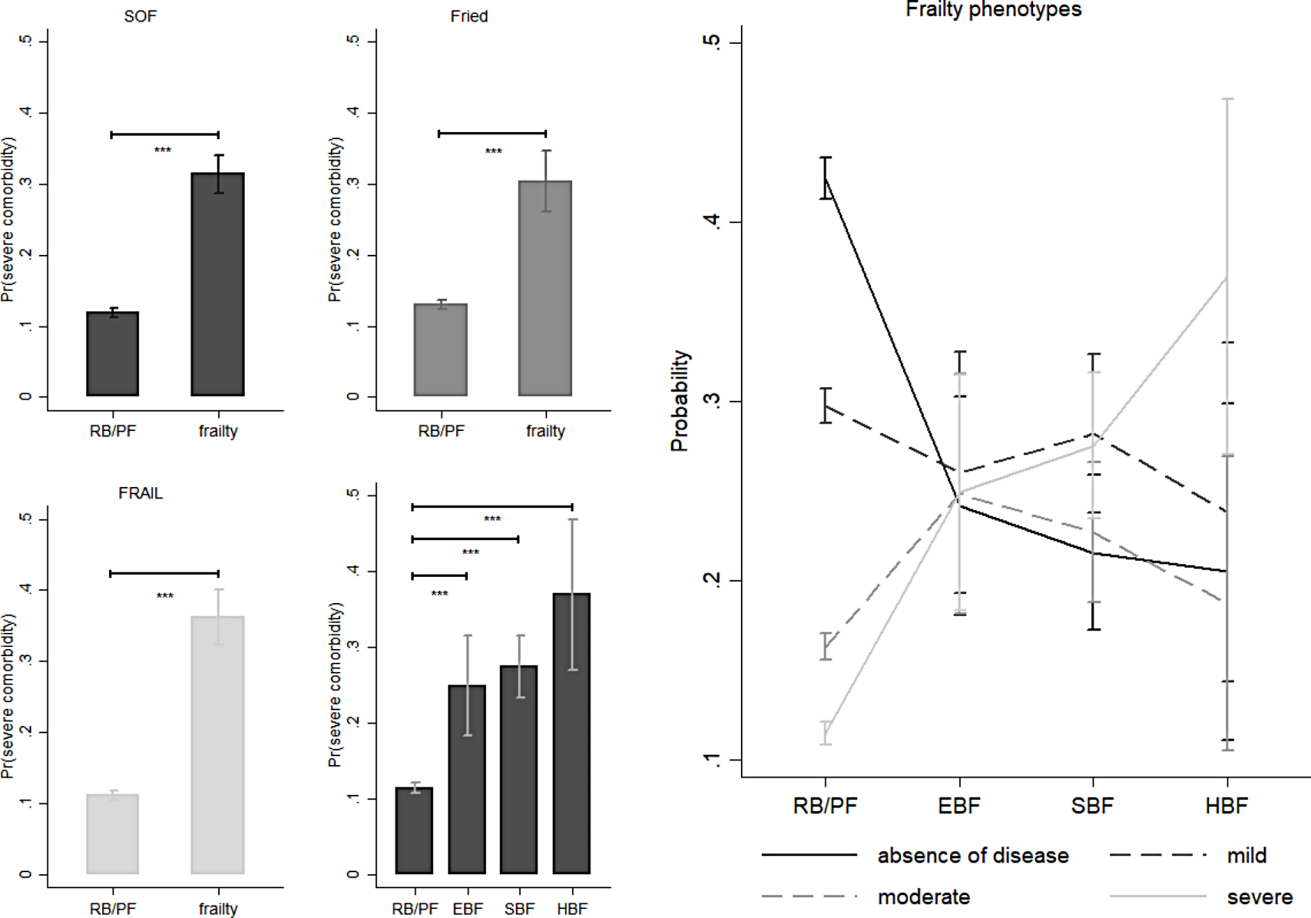
The effects of frailty and frailty phenotypes on the probability of severe comorbidity among middle-aged and older adults, Taiwan, 1996–2007. *Note* All results were based on random-effects panel logit model. The data points represent the mean ± standard error. Pr(severe comorbidity): the predicted probability of severe comorbidity; SOF: the Study of Osteoporotic Fractures index; Fried: the Fried’s frailty index; FRAIL: the Fatigue, Resistance, Ambulation, Illness and Loss of weight index; RB/PF: robust/pre-frailty; EBF: energy-based frailty; SBF: sarcopenia-based frailty; HBF: hybrid-based frailty; ^*^p < 0.05, ^**^p < 0.01, and ^***^p < 0.001. *Source*: the author

**Fig. 4:**
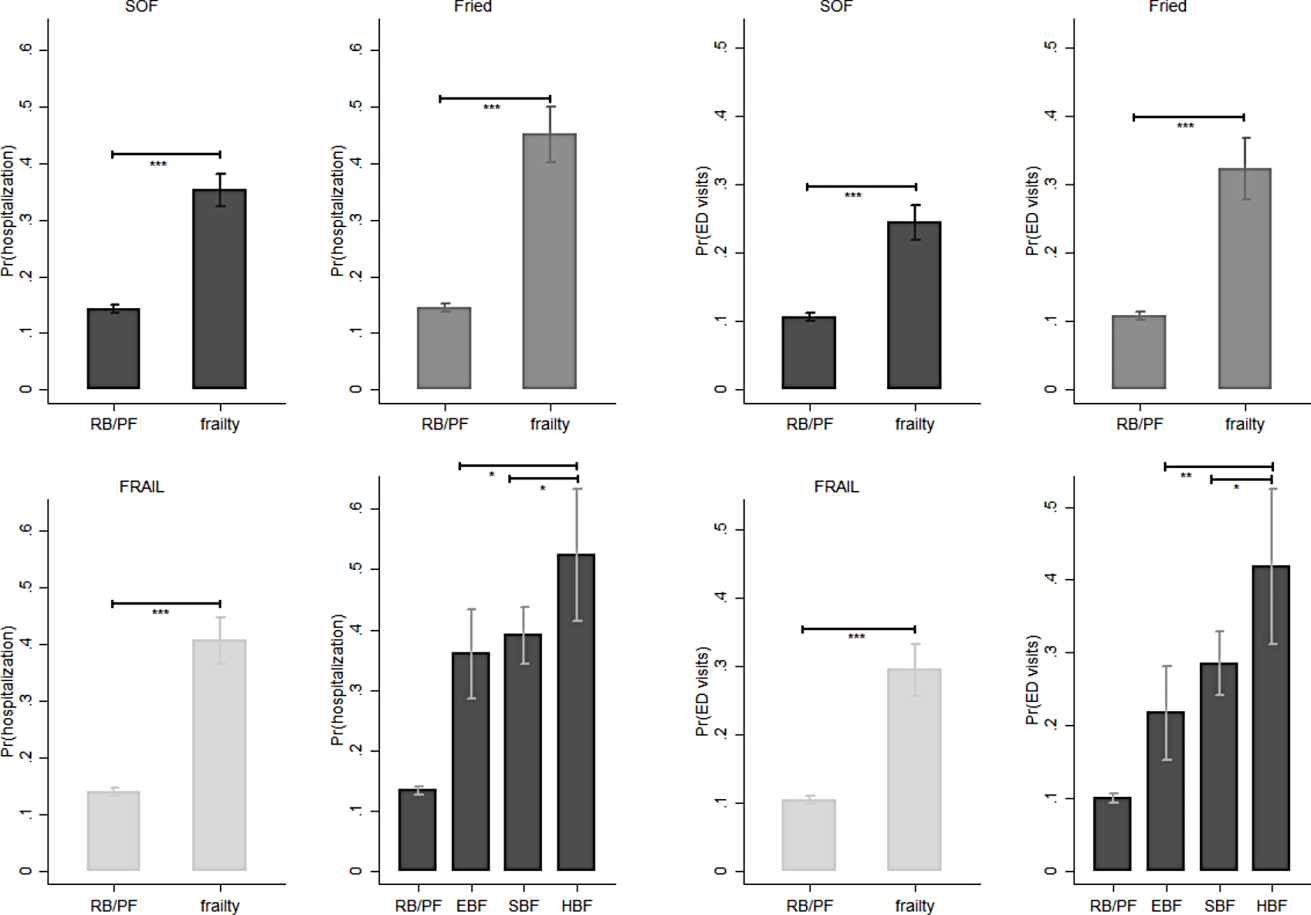
The effects of frailty and frailty phenotypes on the probability of hospitalization and emergency department (ED) visits among middle-aged and older adults, Taiwan, 1996–2007. *Note*: All results were based on random-effects panel logit model. The data points represent the mean ± standard error. Pr(hospitalization): the predicted probability of hospitalization; SOF: the Study of Osteoporotic Fractures index; Fried: the Fried’s frailty index; FRAIL: the Fatigue, Resistance, Ambulation, Illness and Loss of weight index; RB/PF: robust/pre-frailty; EBF: energy-based frailty; SBF: sarcopenia-based frailty; HBF: hybrid-based frailty; ^*^p < 0.05, ^**^p < 0.01, and ^***^p < 0.001. *Source*: the author

**Fig. 5:**
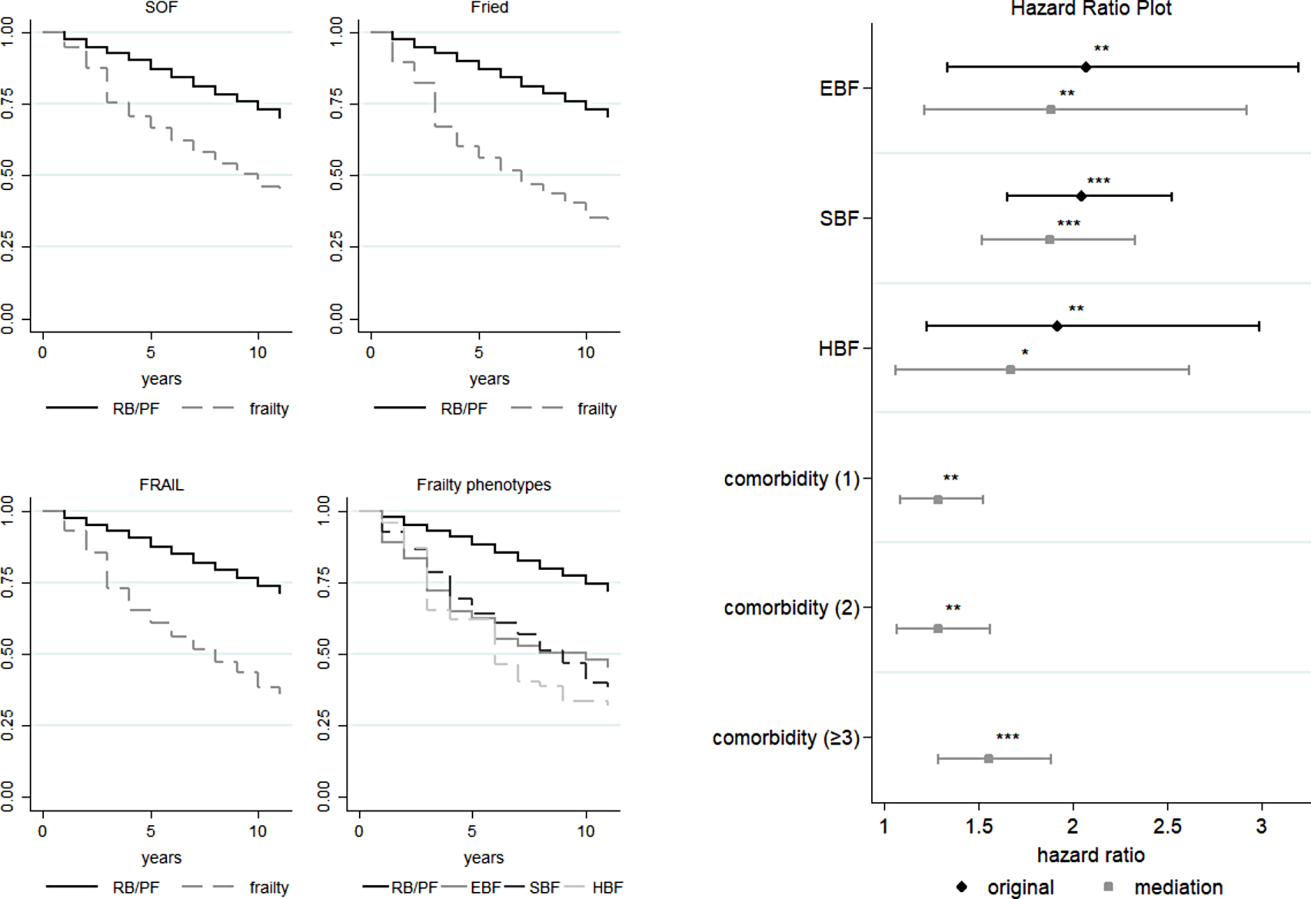
The effects of frailty phenotypes on the risk of mortality among middle-aged and older adults, Taiwan, 1996–2007. *Note* All results were based on the Cox PH model. SOF: the Study of Osteoporotic Fractures index; Fried: the Fried’s frailty index; FRAIL: the Fatigue, Resistance, Ambulation, Illness and Loss of weight index; RB/PF: robust/pre-frailty; EBF: energy-based frailty; SBF: sarcopenia-based frailty; HBF: hybrid-based frailty; Comorbidity (1): mild; Comorbidity (2): moderate; Comorbidity (≥ 3): severe. SOF: Log-rank test: Pr > chi2 = 0.0000; Fried: Log-rank test: Pr > chi2 = 0.0000; FRAIL: Log-rank test: Pr > chi2 = 0.0000; Frailty phenotypes: Log-rank test: Pr > chi2 = 0.0000. Log-rank test between EBF and SBF: Pr > chi2 = 0.4417; Log-rank test between EBF and HBF: Pr > chi2 = 0.3263; Log-rank test between SBF and HBF: Pr > chi2 = 0.8222. ^*^p < 0.05, ^**^p < 0.01, and ^***^p < 0.001. *Source*: the author

The proposed frailty phenotype index was strongly correlated with existing frailty measures. The EBF and SBF indices, which define frailty, pre-frailty, and robustness, were in accordance with those provided by the SOF, FRAIL, and Fried scales, as demonstrated by the chi-square test (Tables S2–S3). Frailty, as defined by SOF, FRAIL, and Fried, was associated with a higher likelihood of hipbone fractures (Fig. 1), depression (Fig. 2), comorbidities (Fig. 3), ED visits or hospital admission (Fig. 4), and mortality (Fig. 5).

Compared with existing frailty measures, there are differences in the health consequences between the frailty phenotypes defined by the EBF and SBF indices. Individuals with only the EBF were found to be at a lower risk of falls and fractures than their counterparts with only the SBF (adjusted odds ratio [AOR] = 0.13, 95% confidence interval [CI] = 0.03–0.46) or with HBF (AOR = 0.21, 95% CI = 0.04–1.03, *p* < 0.1). There were no statistically significant differences in the hip fracture outcomes between older adults with SBF and those with HBF (Fig. 1). By contrast, depression was less likely among those in the SBF only than those in the EBF only (AOR = 0.02, 95% CI = 0.01–0.05) or those with HBF (AOR = 0.04, 95% CI = 0.01–0.12). Furthermore, the results revealed no statistically significant differences in the likelihood of depression between older adults who reported EBF and those who reported HBF (Fig. 2).

The likelihood of comorbidity was not significantly different among the three frailty phenotypes, although they were associated with a higher chance of developing comorbidities than robust health status (e.g., severe comorbidities, EBF: AOR = 7.17, 95% CI = 3.88–13.24; SBF: AOR = 9.80, 95% CI = 6.27–15.30; HBF: AOR = 14.67, 95% CI = 5.57–38.61) (Fig. 3). Hybrid-based frail older adults were more likely to be hospitalized (AOR = 1.84, 95% CI = 1.08–3.14) and have ED visits (AOR = 2.03, 95% CI = 1.15–3.58) than sarcopenia-based frail older adults. When comparing older adults with HBF and those with EBF, these disparities in hospital admissions (AOR = 2.13, 95% CI = 1.16–3.94) and ED visits (AOR = 3.11, 95% CI = 1.57–6.17) become apparent. There were no statistically significant differences in the likelihood of hospital admission or ED visits between energy-based and sarcopenia-based frail older adults (Fig. 4).

Figure 5 reveals the impact of frailty phenotypes on the survival curves of the individuals. Robust and pre-frail older adults survive longer than their counterparts with the three frailty phenotypes. However, the survival curves of the individuals with distinct frailty phenotypes were not significantly different. According to the Cox PH model, individuals with EBF, SBF, and HBF were at a higher risk of mortality (EBF: adjusted hazard ratio [AHR] = 2.06, 95% CI = 1.33–3.20; SBF: AHR = 2.04, 95% CI = 1.65–2.52; HBF: AHR = 1.91, 95% CI = 1.22–2.99) (Fig. 5). There were no statistically significant differences in mortality among the three frailty phenotypes. Following a mediation analysis with survival data, the effect of frailty phenotypes on the risk of mortality was reduced when the explanatory variable and the mediator (comorbidity) were included (EBF: AHR = 1.88, 95% CI = 1.21–2.92; SBF: AHR = 1.88, 95% CI = 1.52–2.33; HBF: AHR = 1.67, 95% CI = 1.06–2.62). However, indirect effects were found to be statistically significant: comorbidity was associated with a higher risk of mortality (e.g., severe comorbidities, AHR = 1.55, 95% CI = 1.28–1.88) (Fig. 5). The results of the robustness tests were in accordance with our primary analysis of the impact of the frailty phenotypes (Table S4).

The study failed to capture the disparities in hipbone fractures when one component of the EBF index was replaced with another component of the SBF index (Table S5). This may be because the revised indices include components that assess muscle function or physical activity. Furthermore, according to the modified frailty index, sarcopenia-based frail older adults were more likely to develop depression than their energy-based frail counterparts, and hybrid-based frail older adults were more depressed than their equivalents in the other two frailty types (Table S6). This may be because, when all modified indices contain components that include each other, older adults deemed to possess a higher proportion of frailty components are more likely to have depression.

## Discussion

Frailty, as defined by the SOF, FRAIL, and Fried, was associated with a higher likelihood of poor health performance. This finding is consistent with existing research indicating an association between frailty and fractures [12, 13], depression [14, 15], comorbidities [16, 17], and ED visits or hospital admissions [18, 19]. However, frailty phenotypes differ in their ability to explain distinct health outcomes.

First, the findings indicate that energy-based frail older adults are likely to report depression, which is congruent with the evidence for the unfavorable effects of weight loss and fatigue on psychological well-being. In the Health and Retirement Study, weight loss was associated with a slight increase in depressive symptoms in men [38]. In the Health, Aging, and Body Composition Study, weight loss over three years predicted a depressed mood in the fourth year [39]. In the English Longitudinal Study of Aging, the weight loss group exhibited a higher level of depressed mood at follow-up compared to the groups that remained weight stable or gained weight. In some of the adjusted analyses, it was observed that the weight loss group had low well-being [40]. Older adults with involuntary weight loss are significantly more likely to experience fatigue, which may be partly due to a lack of energy or nutrients [41]. Fatigue is associated with a greater likelihood of subsequent deterioration in self-rated health, functional status, loneliness, depression, and physical activity levels [42]. Both mobility-related and mental fatigue are associated with depressive symptoms in a stepwise manner, with higher levels of fatigue associated with higher levels of depressive symptoms [43]. EBF causes involuntary weight loss and fatigue, which suggests that it is strongly associated with depressive symptoms.

Second, the findings revealed that sarcopenia-based frail older adults are likely to have hipbone fractures, which is consistent with the evidence for muscle weakness and physical inactivity as risk factors for sustaining hip fractures. Several studies suggest that handgrip strength, a measure of muscular strength, is associated with the risk of fractures [44, 45]. Furthermore, poor walking ability is a crucial predictor of falls [46, 47] and fractures [48]. Activities of daily living (ADLs) and instrumental activities of daily living (IADLs) are essential for independent living, and low muscle strength and walking ability are associated with worsening ADL and IADL [49]. Broader literature has demonstrated that a decline in physical activity level is associated with falls and fractures [50, 51]. This suggests that SBF is strongly associated with fractures, as it is associated with low muscle strength, low walking ability, and low physical activity.

Third, various frailty phenotypes are associated with comorbidities. However, there were no statistically significant differences in the likelihood of comorbidities between distinct frailty phenotypes. This finding may be related to several studies demonstrating that EBF and SBF are associated with comorbidities. A case-control study of patients aged ≥ 60 who visited a geriatric outpatient clinic at a hospital in Bangkok found that those in the involuntary weight loss group had significantly more comorbidities [52]. The Lifelines Cohort Study revealed that the likelihood of experiencing severe and persistent fatigue increased significantly with each additional chronic disease [53]. A nationally representative health survey of Americans aged ≥ 50 found that muscle mass and strength positively relate to comorbid medical conditions [54]. The Swedish National Study on Aging and Care revealed that multimorbidity is associated with a higher risk of impairment in daily living activities [55].

Fourth, hybrid-based frail older adults were more likely to report hospital admissions and ED visits. It is unsurprising, considering that this frailty phenotype combines both the EBF and SBF patterns, which correspond to severe frailty as defined by the existing frailty measures. Indeed, the severe frailty group, as defined by the frailty index, is likely to require hospitalization [18, 19]. A study using cross-sectional data from the Singapore Longitudinal Ageing Studies to develop the frailty risk index (FRI) found that FRI scores at baseline were significantly associated with functional dependency, hospitalization, and combined adverse health outcomes at follow-up [56]. A cohort study based on Taiwan’s National Health Insurance Research Database constructed a multimorbidity frailty index. It categorized the study population into fit, mild frailty, moderate frailty, and severe frailty groups, revealing that older adults with severe frailty had the highest risk of all-cause mortality, hospitalization, and intensive care unit admission [22]. A recent study developed an electronic frailty index based on diagnostic codes, health indicators, and laboratory data and classified patients into four groups. The findings showed that severe frailty was associated with in-hospital mortality, prolonged hospital stay, and hospital readmission [23]. The newly created FI-LAB index revealed that individuals with high or moderate FI-LAB scores had the highest risk of in-hospital mortality, intensive care unit admission, prolonged hospital stay, and hospital readmission [24]. Future research should employ a frailty index that considers accumulation deficits to identify the severe frailty group [22–24] and subsequently compare it with the hybrid-based frailty group to examine whether both groups exhibit a higher risk of hospitalization and ED visits.

Fifth, frailty phenotypes did not exhibit a statistically significant difference in the mortality risk. Mediation analyses revealed that comorbidities mediated the effect of frailty phenotypes on the risk of mortality among older adults, as there was no significant difference in comorbidities among those with one of the three frailty subtypes, which, in turn, resulted in no disparity in the risk of mortality. This suggests a pathway linking frailty phenotypes, comorbidities, and mortality risk among older adults. The mortality curves for those defined as frail according to existing frailty scales (e.g., the Fried’s criteria) are similar to those defined as frail based on their frailty phenotypes. One potential explanation is that the likelihood of comorbidities is not significantly different between those considered frail based on the frailty phenotype and those on existing frailty scales, and comorbidities are linked to mortality. Our findings differ from those of previous studies based on one-wave follow-up, indicating that the frailty subtype characterized by a slower gait and weaker grip strength is associated with a higher risk of comorbidities and mortality [25–27]. This distinction may reflect the fact that this study used panel data to examine the cross-sectional and longitudinal natures of the relationship by assessing the potential evolution of frailty phenotypes over time. The short- and long-term consequences of the frailty phenotypes warrant further investigation.

A valid frailty index should justify the components that should be included and cutoff values that should be used to determine frailty status. This study supports using current thresholds for defining frailty, as the proposed frailty phenotype index was strongly correlated with existing frailty measures. This study demonstrated that the original definition of frailty phenotypes may explain the diverse aspects of health disparities among older adults, since altering the components of the EBF and SBF indices may eliminate the link between frailty phenotypes and specific health outcomes. Future studies should use this method to test the validity of the disparate frailty phenotype indices.

This study advances the frailty research. First, this study categorizes the components of three distinct frailty scales into energy- and sarcopenia-based frailty, thereby distinguishing it from previous studies that solely used the components of a single frailty scale as the basis for classification [25–29]. Second, studies applying existing frailty measures have confirmed that frail older adults have worse health outcomes across many dimensions [12–19]. The proposed frailty phenotype classification differs from the existing frailty measures in its ability to distinguish the corresponding phenotypes underlying various health consequences. Third, studies examining frailty subtypes have confirmed that one type performs worse on all aspects of health outcomes [25–27]. Our proposed frailty phenotypes could explain the differences in various health consequences.

This study had several limitations that warrant further examination. First, the reliance on self-reported data for certain variables may have resulted in a bias. To reduce participants’ memory bias, this study transformed the rates of hospitalization and ED visits to determine whether these events had ever occurred. However, they may exhibit cognitive biases if questioned about their own capabilities. Combining surveys with clinical data facilitates a more objective assessment of the associations between frailty phenotypes and health outcomes. Frailty phenotype components were subjectively assessed, and objective data should be used to evaluate the validity of the index. Future studies should assess resistance by measuring the participant’s ability to rise from a chair five consecutive times without using the arms and assess handgrip strength and walking ability through diagnostic tools.

Second, this study could not use the Charlson Comorbidity Index or Cumulative Illness Rating Scale because not all medical comorbid conditions, whether different clinical weights were used, were available in the TLSA survey. We divided the total number of comorbid conditions that the TLSA could provide at distinct levels according to existing studies that divide it into four levels [57, 58]. Future studies should use validated scales to assess comorbidities.

Third, some participants who completed the questionnaire after 1996 were loss to follow-up. Selection bias because of loss to follow-up threatens the internal validity of estimates derived from longitudinal data. Nevertheless, the inverse probability weighting models that accounted for the participants’ loss to follow-up revealed robust results (Table S7).

Fourth, more detailed biomarker data may enable examination of the mechanisms underlying the impact of frailty phenotypes on diverse health outcomes. A recent study found a correlation between the frailty phenotype and acoustic parameters [59], as the airflow volume initiated by lung contraction and glottal flow stability during phonation may be reflected in the acoustic characteristics, which may be related to an individual’s energy status and muscle control. Future research should examine the mechanisms linking frailty phenotypes and anatomical changes that result in alterations in the acoustic properties of voice to diverse health consequences.

Finally, the findings based on a Taiwanese sample limit generalizability. Future studies should employ other nationally representative samples, such as the Irish Longitudinal Study on Aging and Canadian Longitudinal Study on Aging, to examine whether this conclusion applies in other populations.

## Conclusion

The proposed frailty phenotype may explain the distinct health outcomes. This study has several implications for clinical practice and health policies. First, healthcare professionals can use the proposed frailty phenotype classification to clearly understand ways to intervene and employ more effective approaches to improve the frailty status and mitigate potential adverse health outcomes. Second, governments should develop health policies that address the diverse aspects of adverse health consequences by considering the corresponding frailty phenotypes. Regardless of whether it is a clinical intervention or health management, reducing weight loss and fatigue may be an effective preventive medical strategy for deterring energy-based frailty and subsequent depression in older adults. Improving muscle function and physical activity is a plausible way to alleviate sarcopenia-based frailty and potential fractures. These strategies may promote health development in older adults.

## Electronic supplementary material

Below is the link to the electronic supplementary material.

Supplementary Material 1

## Data Availability

Data are available from the corresponding author upon reasonable request (politicshtyan@gmail.com).
