# Supplementary material for: Frailty phenotypes and their association with health consequences: a comparison of different measures
Source: Aging Clin Exp Res. 2024 Dec 3;36(1):233. doi: 10.1007/s40520-024-02887-4 (PMC11614962; doi:10.1007/s40520-024-02887-4)
Supplement: Supplementary file 1 — Supplementary Material 1 [file 40520_2024_2887_MOESM1_ESM.docx]

**Supplementary Information**

**Table S1** Characteristics of a sample of pooled time series and cross-sectional observations: fractures and depression

|  | Fractures | | p-value | Depression | | p-value |
| --- | --- | --- | --- | --- | --- | --- |
|  | No | Yes |  | No | Yes |  |
| N | 15,901 | 517 |  | 11,942 | 3,111 |  |
| (%) | (96.85) | (3.15) |  | (79.33) | (20.67) |  |
| Age |  |  | <0.001 |  |  | <0.001 |
| 50-64 | 5,077 | 85 |  | 4,188 | 756 |  |
|  | (31.93) | (16.44) |  | (35.07) | (24.30) |  |
| 65-74 | 5,757 | 143 |  | 4,380 | 1,225 |  |
|  | (36.21) | (27.66) |  | (36.68) | (39.38) |  |
| ≥ 75 | 5,067 | 289 |  | 3,374 | 1,130 |  |
|  | (31.87) | (55.90) |  | (28.25) | (36.32) |  |
| Age (continuous) |  |  | <0.001 |  |  | <0.001 |
| Sex |  |  | <0.001 |  |  | <0.001 |
| Female | 7,491 | 314 |  | 5,239 | 1,866 |  |
|  | (47.11) | (60.74) |  | (43.87) | (59.98) |  |
| Male | 8,410 | 203 |  | 6,703 | 1,245 |  |
|  | (52.89) | (39.26) |  | (56.13) | (40.02) |  |
| Education (years) |  |  | <0.001 |  |  | <0.001 |
| 0 | 5,884 | 266 |  | 3,799 | 1,596 |  |
|  | (37.00) | (51.45) |  | (31.81) | (51.30) |  |
| 1-6 | 6,337 | 164 |  | 5,028 | 1,079 |  |
|  | (39.85) | (31.72) |  | (42.10) | (34.68) |  |
| 7-12 | 2,749 | 65 |  | 2,300 | 355 |  |
|  | (17.29) | (12.57) |  | (19.26) | (11.41) |  |
| ≥ 13 | 931 | 22 |  | 815 | 81 |  |
|  | (5.85) | (4.26) |  | (6.82) | (2.60) |  |
| Area of residence |  |  | <0.001 |  |  | 0.012 |
| Rural | 5,489 | 200 |  | 4,087 | 1,119 |  |
|  | (34.52) | (38.68) |  | (34.22) | (35.97) |  |
| Suburban | 3,032 | 124 |  | 2,292 | 635 |  |
|  | (19.07) | (23.98) |  | (19.19) | (20.41) |  |
| Urban | 7,380 | 193 |  | 5,563 | 1,357 |  |
|  | (46.41) | (37.33) |  | (46.58) | (43.62) |  |
| Marital status ^a^ |  |  | <0.001 |  |  | <0.001 |
| Having a spouse | 10,793 | 259 |  | 8,622 | 1,770 |  |
|  | (67.88) | (50.10) |  | (72.21) | (56.89) |  |
| Not having a spouse | 5,106 | 258 |  | 3,319 | 1,341 |  |
|  | (32.12) | (49.90) |  | (27.79) | (43.11) |  |
| Current living status |  |  | 0.776 |  |  | <0.001 |
| Living with spouse, etc. | 14,423 | 471 |  | 10,917 | 2,689 |  |
|  | (90.73) | (91.10) |  | (91.44) | (86.44) |  |
| Living alone | 1,473 | 46 |  | 1,022 | 422 |  |
|  | (9.27) | (8.90) |  | (8.56) | (13.56) |  |
| Smoking status |  |  | 0.001 |  |  | <0.001 |
| Non-smoker | 12,345 | 434 |  | 9,094 | 2,514 |  |
|  | (77.64) | (83.95) |  | (76.15) | (80.81) |  |
| Smoker | 3,556 | 83 |  | 2,848 | 597 |  |
|  | (22.36) | (16.05) |  | (23.85) | (19.19) |  |
| Alcohol intake |  |  | 0.032 |  |  | <0.001 |
| Non-alcohol drinker | 12,211 | 418 |  | 8,784 | 2,628 |  |
|  | (76.80) | (80.85) |  | (73.56) | (84.47) |  |
| Alcohol drinker | 3,688 | 99 |  | 3,157 | 113 |  |
|  | (23.20) | (19.15) |  | (26.44) | (9.89) |  |
| Frequency of exercise |  |  | <0.001 |  |  | <0.001 |
| 0 | 6,535 | 309 |  | 4,225 | 1,661 |  |
|  | (41.11) | (59.77) |  | (35.39) | (53.39) |  |
| 1 | 924 | 29 |  | 718 | 180 |  |
|  | (5.81) | (5.61) |  | (6.01) | (5.79) |  |
| 2 | 1,606 | 41 |  | 1,286 | 292 |  |
|  | (10.10) | (7.93) |  | (10.77) | (9.39) |  |
| ≥ 3 | 6,832 | 138 |  | 5,710 | 978 |  |
|  | (42.98) | (26.69) |  | (47.83) | (31.44) |  |
| EBF |  |  | <0.001 |  |  | <0.001 |
| Robust | 10,863 | 242 |  | 9,609 | 1,468 |  |
|  | (78.04) | (65.58) |  | (84.70) | (50.43) |  |
| Pre-frailty | 2,697 | 108 |  | 1,720 | 1,084 |  |
|  | (19.38) | (29.27) |  | (15.16) | (37.24) |  |
| Frailty | 360 | 19 |  | 16 | 359 |  |
|  | (2.59) | (5.15) |  | (0.14) | (12.33) |  |
| SBF |  |  | <0.001 |  |  | <0.001 |
| Robust | 9,970 | 100 |  | 8,570 | 1,153 |  |
|  | (78.39) | (28.01) |  | (85.73) | (56.11) |  |
| Pre-frailty | 1,758 | 88 |  | 1,181 | 540 |  |
|  | (13.82) | (24.65) |  | (11.81) | (26.28) |  |
| Frailty | 991 | 169 |  | 245 | 362 |  |
|  | (7.79) | (47.34) |  | (2.45) | (17.62) |  |
| SOF |  |  | <0.001 |  |  | <0.001 |
| Robust | 8,251 | 86 |  | 7,546 | 769 |  |
|  | (67.22) | (27.65) |  | (74.23) | (32.24) |  |
| Pre-frailty | 2,978 | 134 |  | 2,231 | 874 |  |
|  | (24.26) | (43.09) |  | (21.95) | (36.65) |  |
| Frailty | 1,046 | 91 |  | 389 | 742 |  |
|  | (8.52) | (29.26) |  | (3.83) | (31.11) |  |
| Fried |  |  | <0.001 |  |  | <0.001 |
| Robust | 9,426 | 150 |  | 8,508 | 1,052 |  |
|  | (75.39) | (53.76) |  | (81.18) | (46.34) |  |
| Pre-frailty | 2,686 | 86 |  | 1,853 | 912 |  |
|  | (21.48) | (30.82) |  | (17.68) | (40.18) |  |
| Frailty | 391 | 43 |  | 120 | 306 |  |
|  | (3.13) | (15.41) |  | (1.14) | (13.48) |  |
| FRAIL |  |  | <0.001 |  |  | <0.001 |
| Robust | 8,522 | 103 |  | 7,844 | 759 |  |
|  | (72.78) | (39.31) |  | (79.59) | (36.47) |  |
| Pre-frailty | 2,633 | 98 |  | 1,838 | 887 |  |
|  | (22.49) | (37.40) |  | (18.65) | (42.62) |  |
| Frailty | 555 | 61 |  | 173 | 435 |  |
|  | (4.74) | (23.28) |  | (1.76) | (20.90) |  |
| Year |  |  | <0.001 |  |  | 0.004 |
| 1996 | 5,027 | 86 |  | 3,670 | 1,040 |  |
|  | (31.61) | (16.63) |  | (30.73) | (33.43) |  |
| 1999 | 4,297 | 112 |  | 3,263 | 837 |  |
|  | (27.02) | (21.66) |  | (27.32) | (26.90) |  |
| 2003 | 3,606 | 161 |  | 2,807 | 649 |  |
|  | (22.68) | (31.14) |  | (23.51) | (20.86) |  |
| 2007 | 2,971 | 158 |  | 2,202 | 585 |  |
|  | (18.68) | (30.56) |  | (18.44) | (18.80) |  |

*Note*: missing values in some variable in the dataset.

^a^ Not having a spouse: Widowed/divorced/separated/never married; Having a spouse: Married or living with a partner.

**Table S2** Comparison of the results of energy-based frailty with those of existing frailty measures

|  | Frailty (EBF) | | | p-value |
| --- | --- | --- | --- | --- |
|  | Robust | Pre-frailty | Frailty |  |
| N | 11,139 | 2,819 | 381 |  |
| (%) | (77.68) | (19.66) | (2.66) |  |
| SOF |  |  |  | <0.001 |
| Robust | 8,358 | 0 | 0 |  |
|  | (84.54) | (0.00) | (0.00) |  |
| Pre-frailty | 1,529 | 1,595 | 0 |  |
|  | (15.46) | (65.83) | (0.00) |  |
| Frailty | 0 | 828 | 316 |  |
|  | (0.00) | (34.17) | (100.00) |  |
| Fried |  |  |  |  |
| Robust | 9,320 | 286 | 0 | <0.001 |
|  | (91.81) | (11.94) | (0.00) |  |
| Pre-frailty | 772 | 1,871 | 140 |  |
|  | (7.61) | (78.09) | (50.36) |  |
| Frailty | 59 | 239 | 138 |  |
|  | (0.58) | (49.64) | (49.64) |  |
| FRAIL |  |  |  | <0.001 |
| Robust | 8,644 | 0 | 0 |  |
|  | (90.41) | (0.00) | (0.00) |  |
| Pre-frailty | 883 | 1,749 | 112 |  |
|  | (9.24) | (80.71) | (40.29) |  |
| Frailty | 34 | 418 | 166 |  |
|  | (0.36) | (19.29) | (59.71) |  |

*Note*: missing values in some variable in the dataset.

**Table S3** Comparison of the results of sarcopenia-based frailty with those of existing frailty measures

|  | Frailty (SBF) | | | p-value |
| --- | --- | --- | --- | --- |
|  | Robust | Pre-frailty | Frailty |  |
| N | 10,098 | 1,856 | 1,168 |  |
| (%) | (76.95) | (14.14) | (8.90) |  |
| SOF |  |  |  | <0.001 |
| Robust | 7,878 | 191 | 2 |  |
|  | (83.90) | (11.88) | (0.38) |  |
| Pre-frailty | 1,410 | 964 | 236 |  |
|  | (15.02) | (59.95) | (44.53) |  |
| Frailty | 102 | 453 | 292 |  |
|  | (1.09) | (28.17) | (55.09) |  |
| Fried |  |  |  |  |
| Robust | 7,878 | 839 | 0 | <0.001 |
|  | (83.90) | (52.18) | (0.00) |  |
| Pre-frailty | 1,512 | 722 | 181 |  |
|  | (16.10) | (44.90) | (34.15) |  |
| Frailty | 102 | 47 | 349 |  |
|  | (0.00) | (2.92) | (65.85) |  |
| FRAIL |  |  |  | <0.001 |
| Robust | 7,522 | 398 | 0 |  |
|  | (83.06) | (34.19) | (0.00) |  |
| Pre-frailty | 1,527 | 608 | 209 |  |
|  | (16.86) | (52.23) | (42.05) |  |
| Frailty | 7 | 158 | 288 |  |
|  | (0.08) | (13.57) | (57.95) |  |

*Note*: missing values in some variable in the dataset.

**Table S4** Robustness check: predictors of frailty phenotypes that may influence health consequences among older adults in Taiwan, 1996–2007

|  | Model 1^a^ | | | | Model 2^a^ | | | | Model 3^a^ | | | |
| --- | --- | --- | --- | --- | --- | --- | --- | --- | --- | --- | --- | --- |
|  | Fractures  (reference=“no”) | | | | Depression  (reference=“no”) | | | | Comorbidity (severe)  (reference=“no”) | | | |
|  | Yes | | | | Yes | | | | Yes | | | |
|  | AOR (95% CI) | | | | AOR (95% CI) | | | | AOR (95% CI) | | | |
| Frailty phenotypes |  |  |  |  |  |  |  |  |  |  |  |  |
| Robust/Pre-frailty | 1.57 | (0.31 | , | 7.86) | 0.002 | (0.001 | , | 0.01)^***^ | 0.14 | (0.08 | , | 0.25)^***^ |
| EBF | REF |  |  |  | REF |  |  |  | REF |  |  |  |
| SBF | 16.94 | (3.12 | , | 91.90)^**b^ | 0.02 | (0.01 | , | 0.04)^***^ | 1.11 | (0.54 | , | 2.29) |
| HBF | 12.26 | (1.80 | , | 83.52)^*^ | 0.45 | (1.11 | , | 1.82) | 1.99 | (0.66 | , | 6.01) |
| Frailty phenotypes |  |  |  |  |  |  |  |  |  |  |  |  |
| Robust/Pre-frailty | 0.09 | (0.05 | , | 0.16)^***^ | 0.14 | (0.11 | , | 0.19)^***^ | 0.13 | (0.08 | , | 0.19)^***^ |
| EBF | 0.06 | (0.01 | , | 0.32)^**^ | 60.16 | (22.85 | , | 158.4)^***^ | 0.90 | (0.44 | , | 1.85) |
| SBF | REF |  |  |  | REF |  |  |  | REF |  |  |  |
| HBF | 0.72 | (0.24 | , | 2.14) | 27.20 | (9.17 | , | 80.69)^***^ | 1.79 | (0.65 | , | 4.91) |

*Note*: AOR, adjusted odds ratio; CI, confidence interval; REF, reference groups. Individual-level characteristics were set as control variables. These included gender, age, education level, area of residence, marital status, current living status, smoking status, alcohol intake, and frequency of exercise. ^a^ The study used an alternative measure to lower limb muscle strength, by asking the participants if they had any difficulty walking up two or three flights of stairs without using aids. ^b +^ *p* < 0.1, ^*^ *p* < 0.05, ^**^ *p* < 0.01, and ^***^ *p* < 0.001.

**Table S4** Robustness check: predictors of frailty phenotypes that may influence health consequences among older adults in Taiwan, 1996–2007 (Continue)

|  | Model 4^a^ | | | | Model 5^a^ | | | | Model 6^a^ | | | |
| --- | --- | --- | --- | --- | --- | --- | --- | --- | --- | --- | --- | --- |
|  | Hospitalization  (reference=“no”) | | | | ED visits  (reference=“no”) | | | | Mortality  (reference=“no”) | | | |
|  | Yes | | | | Yes | | | | Yes | | | |
|  | AOR (95% CI) | | | | AOR (95% CI) | | | | AHR (95% CI) | | | |
| Frailty phenotypes |  |  |  |  |  |  |  |  |  |  |  |  |
| Robust/Pre-frailty | 0.23 | (0.16 | , | 0.33)^***b^ | 0.34 | (0.22 | , | 0.53)^***^ | 0.38 | (0.26 | , | 0.56)^***^ |
| EBF | REF |  |  |  | REF |  |  |  | REF |  |  |  |
| SBF | 1.13 | (0.74 | , | 1.73) | 1.41 | (0.85 | , | 2.33) | 0.80 | (0.52 | , | 1.21) |
| HBF | 2.26 | (1.22 | , | 4.19)^**^ | 3.46 | (1.74 | , | 6.85)^***^ | 0.75 | (0.43 | , | 1.32) |
| Frailty phenotypes |  |  |  |  |  |  |  |  |  |  |  |  |
| Robust/Pre-frailty | 0.20 | (0.16 | , | 0.26)^***^ | 0.24 | (0.18 | , | 0.32)^***^ | 0.48 | (0.39 | , | 0.60)^***^ |
| EBF | 0.89 | (0.58 | , | 1.36) | 0.71 | (0.43 | , | 1.17) | 1.26 | (0.83 | , | 1.91) |
| SBF | REF |  |  |  | REF |  |  |  | REF |  |  |  |
| HBF | 2.00 | (1.17 | , | 3.41)^**^ | 2.46 | (1.39 | , | 4.33)^**^ | 0.94 | (0.60 | , | 1.48) |

*Note*: AOR, adjusted odds ratio; AHR, adjusted hazard ratio; CI, confidence interval; ED, emergency department; REF, reference groups. Individual-level characteristics were set as control variables. These included gender, age, education level, area of residence, marital status, current living status, smoking status, alcohol intake, and frequency of exercise. ^a^ The study used an alternative measure to lower limb muscle strength, by asking the participants if they had any difficulty walking up two or three flights of stairs without using aids. ^b +^ *p* < 0.1, ^*^ *p* < 0.05, ^**^ *p* < 0.01, and ^***^ *p* < 0.001.

**Table S5** Robustness check: predictors of frailty phenotypes that may influence fractures among older adults in Taiwan, 1996–2007

|  | Model 1^a^ | | | | Model 2^b^ | | | | Model 3^c^ | | | | Model 4^d^ | | | | Model 5^e^ | | | |
| --- | --- | --- | --- | --- | --- | --- | --- | --- | --- | --- | --- | --- | --- | --- | --- | --- | --- | --- | --- | --- |
|  | Fractures  (reference=“no”) | | | | Fractures  (reference=“no”) | | | | Fractures  (reference=“no”) | | | | Fractures  (reference=“no”) | | | | Fractures  (reference=“no”) | | | |
|  | Yes | | | | Yes | | | | Yes | | | | Yes | | | | Yes | | | |
|  | AOR (95% CI) | | | | AOR (95% CI) | | | | AOR (95% CI) | | | | AOR (95% CI) | | | | AOR (95% CI) | | | |
| Frailty phenotypes |  |  |  |  |  |  |  |  |  |  |  |  |  |  |  |  |  |  |  |  |
| Robust/Pre-frailty | 0.59 | (0.18 | , | 1.92) | 0.25 | (0.03 | , | 1.99) | 0.33 | (0.19 | , | 0.58)^***^ | 0.35 | (0.18 | , | 0.71)^**^ | 0.17 | (0.07 | , | 0.42)^***^ |
| EBF | REF |  |  |  | REF |  |  |  | REF |  |  |  | REF |  |  |  | REF |  |  |  |
| SBF | 7.90 | (2.16 | , | 28.91)^**f^ | 2.54 | (0.31 | , | 20.99) | 1.74 | (0.68 | , | 4.47) | 1.68 | (0.60 | , | 4.67) | 1.19 | (0.41 | , | 3.42) |
| HBF | 4.84 | (0.97 | , | 24.16)^+^ | 4.87 | (0.50 | , | 47.08) | 2.30 | (0.86 | , | 6.16) | 2.35 | (0.81 | , | 6.87) | 1.47 | (0.44 | , | 4.91) |
| Frailty phenotypes |  |  |  |  |  |  |  |  |  |  |  |  |  |  |  |  |  |  |  |  |
| Robust/Pre-frailty | 0.07 | (0.04 | , | 0.14)^***^ | 0.10 | (0.05 | , | 0.18)^***^ | 0.19 | (0.08 | , | 0.44)^***^ | 0.21 | (0.09 | , | 0.49)^***^ | 0.14 | (0.07 | , | 0.30)^***^ |
| EBF | 0.13 | (0.03 | , | 0.46)^**^ | 0.39 | (0.05 | , | 3.26) | 0.57 | (0.22 | , | 1.47) | 0.60 | (0.21 | , | 1.65) | 0.84 | (0.29 | , | 2.42) |
| SBF | REF |  |  |  | REF |  |  |  | REF |  |  |  | REF |  |  |  | REF |  |  |  |
| HBF | 0.61 | (0.20 | , | 1.92) | 1.92 | (0.70 | , | 5.29) | 1.32 | (0.43 | , | 4.09) | 1.40 | (0.45 | , | 4.38) | 1.23 | (0.43 | , | 3.57) |

*Note*: AOR, adjusted odds ratio; CI, confidence interval; REF, reference groups. Individual-level characteristics were set as control variables. These included gender, age, education level, area of residence, marital status, current living status, smoking status, alcohol intake, and frequency of exercise. ^a^ Original model. ^b^ The EBF index: weight loss and low handgrip strength. ^c^ The EBF index: weight loss and low resistance. ^d^ The EBF index: weight loss and low walking ability. ^e^ The EBF index: weight loss and low physical activity. ^f +^ *p* < 0.1, ^*^ *p* < 0.05, ^**^ *p* < 0.01, and ^***^ *p* < 0.001.

**Table S6** Robustness check: predictors of frailty phenotypes that may influence depression among older adults in Taiwan, 1996–2007

|  | Model 1^a^ | | | | Model 2^b^ | | | | Model 3^c^ | | | | Model 4^d^ | | | | Model 5^e^ | | | |
| --- | --- | --- | --- | --- | --- | --- | --- | --- | --- | --- | --- | --- | --- | --- | --- | --- | --- | --- | --- | --- |
|  | Depression  (reference=“no”) | | | | Depression  (reference=“no”) | | | | Depression  (reference=“no”) | | | | Depression  (reference=“no”) | | | | Depression  (reference=“no”) | | | |
|  | Yes | | | | Yes | | | | Yes | | | | Yes | | | | Yes | | | |
|  | AOR (95% CI) | | | | AOR (95% CI) | | | | AOR (95% CI) | | | | AOR (95% CI) | | | | AOR (95% CI) | | | |
| Frailty phenotypes |  |  |  |  |  |  |  |  |  |  |  |  |  |  |  |  |  |  |  |  |
| Robust/Pre-frailty | 0.003 | (0.001 | , | 0.01)^***f^ | 0.26 | (0.09 | , | 0.76)^*^ | 0.24 | (0.18 | , | 0.31)^***^ | 0.19 | (0.14 | , | 0.28)^***^ | 0.13 | (0.08 | , | 0.20)^***^ |
| EBF | REF |  |  |  | REF |  |  |  | REF |  |  |  | REF |  |  |  | REF |  |  |  |
| SBF | 0.02 | (0.01 | , | 0.05)^***^ | 2.88 | (0.96 | , | 8.60)^+^ | 3.83 | (2.21 | , | 6.66)^***^ | 3.54 | (1.98 | , | 6.31)^***^ | 2.32 | (1.24 | , | 4.32)^**^ |
| HBF | 0.54 | (0.14 | , | 2.13) | 7.25 | (2.04 | , | 25.75)^**^ | 7.85 | (4.03 | , | 15.29)^***^ | 6.27 | (3.11 | , | 12.64)^***^ | 4.35 | (1.99 | , | 9.54)^***^ |
| Frailty phenotypes |  |  |  |  |  |  |  |  |  |  |  |  |  |  |  |  |  |  |  |  |
| Robust/Pre-frailty | 0.14 | (0.10 | , | 0.18)^***^ | 0.09 | (0.07 | , | 0.12)^***^ | 0.06 | (0.04 | , | 0.10)^***^ | 0.05 | (0.03 | , | 0.09)^***^ | 0.05 | (0.04 | , | 0.08)^***^ |
| EBF | 46.18 | (18.32 | , | 116.4)^***^ | 0.35 | (0.12 | , | 1.04)^+^ | 0.26 | (0.15 | , | 0.45)^***^ | 0.28 | (0.16 | , | 0.50)^***^ | 0.43 | (0.23 | , | 0.81)^**^ |
| SBF | REF |  |  |  | REF |  |  |  | REF |  |  |  | REF |  |  |  | REF |  |  |  |
| HBF | 24.96 | (8.34 | , | 74.64)^***^ | 2.52 | (1.21 | , | 5.26)^*^ | 2.05 | (0.95 | , | 4.43)^+^ | 1.77 | (0.83 | , | 3.80) | 1.88 | (0.90 | , | 3.93)^+^ |

*Note*: AOR, adjusted odds ratio; CI, confidence interval; REF, reference groups. Individual-level characteristics were set as control variables. These included gender, age, education level, area of residence, marital status, current living status, smoking status, alcohol intake, and frequency of exercise. ^a^ Original model. ^b^ The EBF index: weight loss and low handgrip strength. ^c^ The EBF index: weight loss and low resistance. ^d^ The EBF index: weight loss and low walking ability. ^e^ The EBF index: weight loss and low physical activity. ^f +^ *p* < 0.1, ^*^ *p* < 0.05, ^**^ *p* < 0.01, and ^***^ *p* < 0.001.

**Table S7** Inverse probability weighting models: predictors of frailty phenotypes that may influence fractures among older adults in Taiwan, 1996–2007

|  | Model 1 | | | | Model 2 | | | | Model 3 | | | | Model 4 | | | | Model 5 | | | |
| --- | --- | --- | --- | --- | --- | --- | --- | --- | --- | --- | --- | --- | --- | --- | --- | --- | --- | --- | --- | --- |
|  | Fractures  (reference=“no”) | | | | Depression  (reference=“no”) | | | | Comorbidity (severe)  (reference=“no”) | | | | Hospitalization  (reference=“no”) | | | | ED visits  (reference=“no”) | | | |
|  | Yes | | | | Yes | | | | Yes | | | | Yes | | | | Yes | | | |
|  | AOR (95% CI) | | | | AOR (95% CI) | | | | AOR (95% CI) | | | | AOR (95% CI) | | | | AOR (95% CI) | | | |
| Frailty phenotypes |  |  |  |  |  |  |  |  |  |  |  |  |  |  |  |  |  |  |  |  |
| Robust/Pre-frailty | 0.47 | (0.19 | , | 1.15) | 0.01 | (0.003 | , | 0.02)^***^ | 0.36 | (0.25 | , | 0.52)^***^ | 0.25 | (0.18 | , | 0.35)^***^ | 0.34 | (0.23 | , | 0.50)^***^ |
| EBF | REF |  |  |  | REF |  |  |  | REF |  |  |  | REF |  |  |  | REF |  |  |  |
| SBF | 3.08 | (1.17 | , | 8.10)^*a^ | 0.04 | (0.02 | , | 0.09)^***^ | 1.14 | (0.74 | , | 1.76) | 1.13 | (0.76 | , | 1.68) | 1.37 | (0.89 | , | 2.10) |
| HBF | 2.47 | (0.75 | , | 8.18) | 0.50 | (0.13 | , | 1.86) | 1.70 | (0.93 | , | 3.09) | 1.90 | (1.06 | , | 3.41)^*^ | 2.56 | (1.39 | , | 4.72)^**^ |
| Frailty phenotypes |  |  |  |  |  |  |  |  |  |  |  |  |  |  |  |  |  |  |  |  |
| Robust/Pre-frailty | 0.15 | (0.10 | , | 0.23)^***^ | 0.20 | (0.16 | , | 0.26)^***^ | 0.31 | (0.25 | , | 0.40)^***^ | 0.22 | (0.18 | , | 0.28)^***^ | 0.25 | (0.20 | , | 0.32)^***^ |
| EBF | 0.32 | (0.12 | , | 0.86)^*^ | 26.66 | (11.28 | , | 63.01)^***^ | 0.88 | (0.57 | , | 1.36) | 0.88 | (0.59 | , | 1.31) | 0.73 | (0.48 | , | 1.13) |
| SBF | REF |  |  |  | REF |  |  |  | REF |  |  |  | REF |  |  |  | REF |  |  |  |
| HBF | 0.80 | (0.36 | , | 1.81) | 13.16 | (4.59 | , | 37.73)^***^ | 1.49 | (0.91 | , | 2.44) | 1.68 | (1.01 | , | 2.80)^*^ | 1.87 | (1.12 | , | 3.14)^*^ |

*Note*: AOR, adjusted odds ratio; CI, confidence interval; REF, reference groups. Individual-level characteristics were set as control variables. These included gender, age, education level, area of residence, marital status, current living status, smoking status, alcohol intake, and frequency of exercise. ^a *^ *p* < 0.05, ^**^ *p* < 0.01, and ^***^ *p* < 0.001.
